# Determinants for receiving acupuncture for LBP and associated treatments: a prospective cohort study

**DOI:** 10.1186/1472-6963-6-149

**Published:** 2006-11-17

**Authors:** Jean-François Chenot, Annette Becker, Corinna Leonhardt, Stefan Keller, Norbert Donner-Banzhoff, Erika Baum, Michael Pfingsten, Jan Hildebrandt, Michael M Kochen, Heinz-Dieter Basler

**Affiliations:** 1 Dpt. of General Practice, University of Göttingen, Humboldtallee 38, 37073 Goettingen, Germany; 2 Dpt. of General Practice, Preventive and Rehabilitation Medicine, University of Marburg, Robert-Koch-Str. 5, 35033 Marburg, Germany; 3 Institute for Medical Psychology, University of Marburg, Bunsenstr. 3, 35037 Marburg, Germany; 4 Dpt. of Public Health Sciences, University of Hawaii at Manoa, 1960 East-West Rd., Honolulu, HI 96822, USA; 5 Dpt. of Anesthesiology, Pain Clinic, University of Göttingen, Robert-Koch-Str. 40, 37075 Göttingen, Germany

## Abstract

**Background:**

Acupuncture is a frequently used but controversial adjunct to the treatment of chronic low back pain (LBP). Acupuncture is now considered to be effective for chronic LBP and health care systems are pressured to make a decision whether or not acupuncture should be covered. It has been suggested that providing such services might reduce the use of other health care services. Therefore, we explored factors associated with acupuncture treatment for LBP and the relation of acupuncture with other health care services.

**Methods:**

This is a post hoc analysis of a longitudinal prospective cohort study. General practitioners (GPs) recruited consecutive adult patients with LBP. Data on physical function, subjective mood and utilization of health care services was collected at the first consultation and at follow-up telephone interviews for a period of twelve months.

**Results:**

A total of 179 (13 %) out of 1,345 patients received acupuncture treatment. The majority of those (59 %) had chronic LBP. Women and elderly patients were more likely to be given acupuncture. Additional determinants of acupuncture therapy were low functional capacity and chronicity of pain. Chronic (vs. acute) back pain OR 1.6 (CL 1.4–2.9) was the only significant disease-related factor associated with the treatment. The strongest predictors for receiving acupuncture were consultation with a GP who offers acupuncture OR 3.5 (CL 2.9–4.1) and consultation with a specialist OR 2.1 (CL 1.9–2.3). After adjustment for patient characteristics, acupuncture remained associated with higher consultation rates and an increased use of other health care services like physiotherapy.

**Conclusion:**

Receiving acupuncture for LBP depends mostly on the availability of the treatment. It is associated with increased use of other health services even after adjustment for patient characteristics. In our study, we found that receiving acupuncture does not offset the use of other health care resources. A significant proportion of patients who received did not meet the so far only known selection criterion (chonicity). Acupuncture therapy might be a reflection of helplessness in both patients and health care providers.

## Background

Low back pain (LBP) is a major health problem in industrialized countries with significant economical impact [1]. Acupuncture is a popular but controversial alternative treatment option for LBP with few associated adverse side effects [2]. Results of randomised studies of acupuncture for low back pain have been inconclusive due to poor methodological quality and insufficient acupuncture techniques [3,4]. A systematic review and a meta-analysis corroborate a lack of evidence for the treatment of acute LBP. However in chronic LBP acupuncture seems to be a useful adjunct to conventional treatment [5,6].

Many health care systems and health insurances will have to make a decision about whether or not acupuncture for low back pain will be covered. In Germany, acupuncture for LBP was not regularly covered by the statutory health insurance until March 2006. Despite having no definite proof of the effectiveness of acupuncture so far, it is used by more than 20,000 doctors in the country [7]. Pressure from patients and doctors alike to integrate and reimburse acupuncture within the insurance system resulted in the "German Acupuncture Studies Initiative" which is still going on [8-10]. Within the framework of this initiative, registered physicians who completed an accredited training in acupuncture, received privileges to bill sponsoring insurance providers for acupuncture. Patients receiving acupuncture through this initiative were only required to make a co-payment of 10 %. More often, acupuncture is offered as individual health service and billed entirely to the patients. These economic factors might have an influence on who gets acupuncture apart from disease related factors.

A general problem of interventions for LBP is the rather small therapeutic effectivness. This has been attributed to the inability to differentiate subtypes of LBP and select those who are most likely to benefit from a specific intervention [11]. Acupuncture is usually an adjunct treatment combined with other treatment options. A British pre-post-comparison attributed a reduction in 86 % of physiotherapy referrals and 51 % of specialist referrals to the introduction of acupuncture [12]. A British randomized controlled trial (RCT) comparing acupuncture for LBP to standard treatment found acupuncture to be cost effective with regard to pain reduction. Nevertheless, the overall costs were higher, despite some savings due to the offset of some other care resources [13,14]. A recent German RCT observed marked clinical improvement with acupuncture and concluded that acupuncture is relatively cost-effective with regard to quality of life [15]. Consequently, despite its uncertain effectiveness, acupuncture may be a beneficial alternative for economical reasons or have positive effects on quality of life.

The aim of this study was to identify socioeconomic, disease-related and provider-related factors associated with receiving acupuncture for LBP. We also wanted to explore which treatment options are combined with acupuncture, the frequency of health care utilisation and if receiving acupuncture results in the reduced use of other health care services over a period of one year in a large cohort study. Another important question is if physicians follow the limited evidence in favour of using acupuncture for chronic conditions compared with acute LBP.

## Methods

### Study design

This longitudinal prospective cohort study was embedded within a three armed RCT with an educational intervention in a primary care setting [16]. The cohort encompasses all patients enrolled in the trial. The primary goal of the RCT was to asses the impact of treatment on functional capacity. A predefined secondary goal of the study was to explore the variation of health care services for LBP. The intervention consisted in quality circles for general practitioners (GPs) on an evidence-based LBP guideline (in both intervention arms) and in training of practice nurses in motivational counselling to promote physical activity (in one intervention arm). The guideline is in accordance with the European guidelines [17,18]. These guidelines do not advocate acupuncture for LBP, but rather consider the treatment method as a second line intervention with uncertain effectiveness. The study was conducted in two centres (Marburg, Göttingen). Ethical approval was obtained from both study sites.

### General practitioners

The goal was to recruit 120 practices. In all practices, both GPs and practice nurses had to agree to participate in the educational intervention, in case they would be randomized to the intervention arm with motivational counselling. We contacted 818 general practices surrounding the study centres. Addresses were obtained from local health authorities. From 118 practices who agreed to participate, 2 dropped out after randomization. The GPs were on average 12.7 years in practice (Range 1 to 31 years), the average age was 48 years (SD ± 6) (national average 50.4 years) and 42 % of them were female (national average 36 %). A total of 68 (59 %) practices were run by a single GP. The basic demographic data of our sample is not meaningfully different from the national average [19]. Of the 116 participating practices, 25 (21 %) offered acupuncture treatment to their patients.

### Patients

During the recruitment period of 8 weeks, practice nurses asked every patient with LBP to participate in the study and registered these individuals in order to estimate the number of screened patients. Patients were recruited form November 2002 to March 2003. Inclusion criteria were (1) consulting for LBP in general practice, (2) age above 18, (3) ability to read and understand German and (4) written consent.

### Instruments and data collection

After written consent was obtained, baseline socio-demographic data was collected prior to the consultation with a baseline questionnaire. In addition, patients mailed a questionnaire filled in at home to the study centres. During the consultation, GPs assessed warning signs for complicated LBP ("red flags"). At follow-ups 4 weeks, 6 months and 12 months later, study nurses conducted standardised telephone interviews and patients were asked about health care utilization.

The Hanover Functional Ability Questionnaire (HFAQ) was used for the assessment of functional capacity. The HFAQ is a frequently used instrument for the assessment of back pain disability and a scale with good psychometric properties. It consists of 12 items in which patients can rate their limitation in day-to-day functional abilities. It can be compared to the Roland & Morris Scale, but is advantageous in telephone interviews [20]. The scale ranges from 0 (extreme functional limitation) to 100 (no functional limitation), scores below 70 are considered to be a significant impairment.

In order to classify the natural history of LBP, we used a modification of the von Korff procedure as follows [21]:

▪ **Acute LBP **single episode of LBP of less than 90 days duration

▪ **Recurrent LBP **multiple episodes LBP of less then 90 days duration within the last 12 months

▪ **Chronic LBP **more then 90 consecutive days of LBP within the last 12 months.

To estimate the proportion of patients with radicular symptoms, we relied on the reported level of pain radiation into the leg. We considered radiation below the knee as an indicator of possible nerve root irritation. This is a frequently used and pragmatic approach given the absence of reliable methods for assessing radicular pain in large cohorts [22].

For assessment of depression, we applied the German version of the Centre for Epidemiologic Studies Depression Scale (CES-D) [23]. Scores above 23 are considered clinically relevant [24].

In the telephone interview, we also collected data on health care utilisation, e.g. specialist visits, medication, and non-pharmacological treatment for LBP within the last 6 months. In the interview, the study nurses actively presented a list of 42 possible interventions for LBP. They were trained in conducting standardized interviews and able to describe each method in more detail if necessary. Acupuncture sessions were not considered as consultations. We asked them whether they had received injections in the back or in the buttocks. Injections in the back most likely indicate the use of local anaesthetics with or without steroids, injections in the buttocks most likely indicate the application of an analgesic medication.

### Statistical analysis

In a first step, we conducted univariate analyses in order to compare patients who received acupuncture with those who did not. In case of missing data, we provide the number of subjects analysed. Dependent on the biometric properties of the scales, we either used chi^2^-tests for categorical data, or t-tests and non-parametric tests (Kruskall-Wallis) for continuous data. We considered data to be statistically significant at a 5 % level. A calculation of odds ratios allowed to depict 95 % confidence intervals (CI). When data for health care utilization was missing, it was assumed that patients did not receive that particular service.

In a second step, we performed logistic regression analyses. This procedure calculates the probability of receiving or using a specific health service in relation to acupuncture. Only those factors were incorporated into the model that had proved to be significant in previous univariate tests (chronicity, duration of pain, CES-D score, radiation of pain below the knee, baseline HFAQ). Continuous data were dichotomised. For depression we used a cut-off score of >23 and for functional capacity (HFAQ) a cut-off score of > 70. Due to missing data, the models included only 1320 (98 %) patients.

Given the fact that the present study was an embedded cohort study, we also checked if one of the study arms was a significant factor in the model, which was not the case.

Comparison of consultation frequencies and duration of sick leave were adjusted with ANCOVA including the same covariates as the logistic regression models.

In a final step, we calculated a comprehensive model to predict the probability of receiving acupuncture. This model included all of the factors that had demonstrated their significance in previous univariate analyses. This model included only 1163 (87 %) subjects due to list wise deletion. The software package SAS 9.1 was used for analysis.

## Results

### Patients

**Figure 1:**
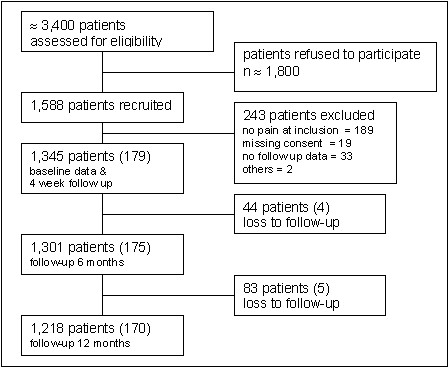
Patient flow (in brackets those who received acupuncture).

Only 60 % of the practices returned registration lists filled in correctly. From this data, it is estimated that approximately 3,400 patients with LBP had been invited to participate. Of those 1,588 patients who agreed to participate, 1,345 were finally included. Reasons for exclusion are listed in Figure 1. The participating 116 practices recruited on average 11.6 (SD ± 5.8) consecutive patients over a period of two months. A total of 1,218 patients were followed up for one year. Overall, 127 patients (9.4 %) dropped out of the study. During the one-year follow-up, 179 (13 %) participants received acupuncture for LBP, 97 at one period, 63 patients at two periods and 18 patients during the entire follow-up. The number of acupuncture sessions ranged from 1 to 47, mean 12 (SD ± 8.8).

### Socioeconomic baseline data

Patients receiving acupuncture tended to be female, slightly older than average and more often retired, while income and education and living with a partner were not associated with the treatment (Table 1).

### Disease-related data

Patients receiving acupuncture had a lower functional capacity at baseline, a longer duration of pain on average and were more likely to experience pain radiating below the knee. The majority (59 %) were classified as chronic back pain sufferers. In addition, they more frequently showed clinically significant depression scores. The presence of "red flags" was not associated with acupuncture (Table 2).

### Health service utilisation

Patients receiving acupuncture had a significantly higher adjusted consultation rate both for GPs and for specialists. Regarding GPs, their rate was 15.6 (CI 13.8–17.6) visits a year versus 9.5 (CI 8.7–10.2) visits a year (p < .001). Regarding specialists, the rate was 13.1 (CI 11.6–14.5)/year versus 3.5 (CI 3–4.2)/year (p < .001). They were OR 3.2 (CI 2–5.2) times more likely to consult a specialist. More then 90 % of the specialists were orthopaedic surgeons. Those acupuncture patients who were part of the work force had not been more frequently on sick-leave after adjustment, but for a significantly longer period of time: 31.8 (CI 23–40.1) days a year versus 16.1 (CI 12.9–19.4) days a year after adjustment (p < .001).

Acupuncture patients received prescriptions for medication, physiotherapy and other treatment modalities like injection therapies more often than non-acupuncture patients. Non-opioid medications mostly consisted of non-steroidal analgesics and opioids were mostly of low potency. The increased use of health services remained significant after adjustment for gender, age, pain chronicity, functional capacity, depression scores and radiation of pain below the knee except for electrotherapy and psychotherapy (Table 3).

### Prediction model for receiving acupuncture

Those GPs who offered acupuncture had recruited 280 patients altogether. 66 (23,6%) of them had actually received this treatment (OR 2.7 (CI 1.9–3.8)). Most of the acupuncture patients (n = 110, 62 % of them), however, had been given the treatment by specialists, the majority of them orthopaedic surgeons.

To explore the role of provider related factors for the provision of acupuncture, we included two variables in a logistic regression model: (1) consulting one of the GPs or (2) consulting one of the specialists that provided acupuncture service. We controlled for socio-demographic and disease-related factors that had been proven to be associated with acupuncture in previous univariate analyses. The results show that consulting such a GP (OR 3.5 (CI 2.9–4.1)), consulting such a specialist (OR 2.1 (CI 1.9–2.3)), and the state of pain chronicity (OR 1.6 (CI 1.4–2.9)) were the only factors significantly associated with receiving acupuncture. Gender, age, radiation of pain, functional capacity, and depression did not contribute to explaining determinants of the treatment.

## Discussion

### Summary of main findings

Our study supports the hypothesis that receiving acupuncture for LBP seems to be rather a function of availability and to a lesser degree dependant on disease-related factors (like pain chronicity). In univariate analyses, patients receiving acupuncture tended to be older and therefore more often retired. They were more likely to be female, while income and education did not predict the application of the treatment. The majority of patients receiving acupuncture also had chronic LBP. In about one third of our sample, acupuncture was probably inappropriate due to the lack of evidence of effectiveness for acute and recurrent LBP. After adjustment for those baseline differences that may partly explain the increased use of health care services for the acupuncture group, acupuncture remained associated with an increased frequency of consultations and of other therapies for LBP.

### Strengths and limitations

This is to date the largest prospective cohort study in Germany that provides clinical data in a population of patients with LBP in this country. Due to the fact that practice nurses also had to agree to participate in the trial, we anticipated a relatively low practice recruitment rate. The sample size and the demographic baseline data of participating GPs make us confident that the data we collected is representative of current clinical practice in Germany. Unfortunately, we do not know how many patients were offered acupuncture and how many of them declined to accept the treatment e.g. due to co-payment or other reasons. Since we do not know if acupuncture was given by the GP or a specialist, we might overestimate the proportion of GPs who contributed to a patient's receiving acupuncture. Nevertheless, a proportion of 13 % of subjects receiving acupuncture is remarkable in a primary care setting for an intervention not covered by the statutory health insurance. Another limitation is that we do not know which method was used for acupuncture and the qualification of the providers. Acupuncture is not a standardized method and may have been applied in various ways. One should keep in mind that the study is a post hoc analysis and was not designed to assess the effectiveness of acupuncture.

### Comparison with other studies

Our findings that females and patients with chronic LBP were more likely to receive acupuncture are not surprising. From other surveys, it is known that women are more prone to try acupuncture [25]. Being male and being in a state of good health was found to be associated with a disregard of complementary alternative medicine (CAM) [26]. In the United Kingdom, higher income was associated with use of CAM [27]. This relation obviously depends on access and coverage provided by the health care system. Since co-payment for acupuncture was required in Germany, we expected low income or unemployment to be associated with less acupuncture, which – contrary to our expectation – was not the case. This could partly be due to the reason that some patients participated in the afore mentioned acupuncture initiative. Ample time and the willingness to adhere to repeated treatment sessions may also be an issue, since retired individuals were more likely to receive acupuncture. Although these factors contribute to explain the participation in acupuncture treatment, there may be other factors we were not able to explore in this study, like previous experience with acupuncture, dissatisfaction with conventional treatment and comorbid conditions.

It has been argued that offering acupuncture services reduces the need for other services like e.g. physiotherapy [12]. The above-mentioned randomized controlled trials found acupuncture to be cost effective partly due to the reduced use of other health care services [12,13]. Our data suggests that receiving acupuncture does not offset the use other health care resources. Contradictory, in our study, acupuncture was associated with increased GP consultation rates, specialist consultations, and prescriptions for physiotherapy. Acupuncture patients were also more likely to receive other (controversial) treatment options like manual therapy, transcutaneous electric nerve stimulation (TENS) and injection therapies. These treatments are considered to be ineffective or of unproven effectiveness and are not recommended by evidence-based guidelines [16,17].

Patients with disabling, recurrent or chronic LBP are more likely to consult a specialist and to need services like pain medication or physiotherapy. The association between an increased use of health care resources and acupuncture is only partly explained by the disease-related factors we collected. Receiving acupuncture seems to be a surrogate parameter for high health service utilisation and might reflect a feeling of helplessness in both patients and health care providers. Nevertheless, the most important factor determining acupuncture treatment is consulting a GP who offers the treatment or a specialist.

## Conclusion

Acupuncture is increasingly accepted as an adjunct to the treatment for LBP. There is so far only one evidence-based selection criterion for prescribing acupuncture, chronicity, which is not well respected. Our study suggests that acupuncture is most frequently used in combination with other either alternative or conventional treatment options. Receiving acupuncture seems to be rather an additional measure for individuals with multiple treatments and unsatisfactory outcomes than a judiciously selected treatment option. Future research of high quality will have to identify those individuals who benefit from acupuncture treatment and will have to clarify which combination of treatment options shows the most beneficial effects.

## Abbreviations

**CAM: **complementary alternative medicine, **CI**: 95 % confidence interval, **GP**: general practitioner, **LBP**: low back pain, **OR**: odds ratio, **RCT**: randomised controlled trial

## Competing interests

The author(s) declare that they have no competing interests.

## Authors' contributions

All authors contributed to the study design. AB, EB, CL, JFC, JH, MP and NDB contributed to data collection. JFC, AB, SK and CL wrote the analysis plan, JFC, AB and CL analysed data. JFC and HDB were the principal authors of the paper. JFC had full access to all data, and is guarantor. All authors contributed to manuscript drafting and revision and approved the final manuscript.

## Pre-publication history

The pre-publication history for this paper can be accessed here:



## Figures and Tables

**Table 1:** Baseline sociodemographic characteristics comparing those who did or did not receive acupuncture for low back pain.

**Sociodemographic data**	**No acupuncture **(n = 1,166)	**Acupuncture **(n = 179)	**p-value**
Age	48.5 (CI 47–49)	51 (CI 49–53)	< .01
Age in age groups (n = 1,199)			
< 40 years	308 (30 %)	40 (24 %)	< .02
40–60 years	514 (50 %)	78 (48 %)	
> 60 years	210 (20 %)	49 (29%)	
Gender			
Female	665 (57%)	116 (65 %)	< .05
Male	501 (43 %)	63 (35 %)	
Body mass index (n = 1,246)	26.7 (CI 26–27)	26.8 (CI 26–27)	n.s.
**School education **(n = 1,191)			
< 10 years	428 (42 %)	72 (44 %)	n.s
10 years	318 (31 %)	60 (36 %))	
> 10 years	256 (25 %)	29 (18 %)	
other	24 (2 %)	4 (2 %)	
Living with partner (n = 1,281)	783 (76 %)	121 (73 %)	n.s.
**Employment status **(n = 1,193)			
▪ Working full or part-time	677 (66 %)	89 (54 %)	< .01
▪ Housekeeping	102 (10 %)	17 (10 %)	
▪ Retired	203 (20 %)	52 (31 %)	
▪ Unemployed	45 (4 %)	8 (5 %)	
**Net income **(n = 1,052)			
▪ < 1,000	164 (18 %)	27 (18 %)	n.s.
▪ 1,001–2,000 Euro	397 (43 %)	77 (50 %)	
▪ 2,001–3,000 Euro	248 (27 %)	38 (25 %)	
▪ >3,000 Euro	108 (12 %)	11 (7 %)	

Table legend text

CI: 95 % confidence intervals

**Table 2:** Disease related data comparing those who did or did not receive acupuncture for low back pain.

**Disease related data ** n = 1345	**No acupuncture ** n = 1,166	**Acupuncture ** n = 179	**p-value**
Severity of pain at baseline (scale 1–10) (n = 1,310)	5.2 (CI 5–5,3)	5.5 (CI 5.1–5.8)	n.s.
Chronicity of LBP			
Acute LBP	238 (21%)	18 (10 %)	< .001
Recurrent LBP	482 (41 %)	56 (31 %)	
Chronic LBP	446 (38 %)	105 (59 %)	
Average duration of the actual pain episode at inclusion in days	60.4 (CI 53–67)	96.7 (CI 74–118)	< .02
Radiation of pain below the knee (n = 1,320)	217 (18 %)	47 (26 %)	< .01
Positive depression-score (CESD) at baseline (n = 1,320)	161 (17 %)	37 (24 %)	< .02.
Suspicion of red flags at baseline	102 (8 %)	16 (9 %)	n.s.
Patients complaining about back pain after 1 year (n = 1,219)	467 (45 %)	106 (62 %)	< .001
(HFAQ) functional capacity at baseline	68.2 (SD ± 21)	62.7 (SD ± 20)	< .01

Table legend text

CI: 95 % confidence intervals

SD: standard deviation

HFAQ: Hanover Functional Disability Questionnaire

**Table 3:** Health service utilisation comparing those who did or did not receive acupuncture for low back pain.

**Health service utilisation**	**No acupuncture** n = 1,145	**Acupuncture** n = 175	**Unadjusted OR**	Adjusted OR*
Specialist consultations	641 (54 %)	150 (84%)	4.5 (CI 3–6.8)	3.2 (CI 2–5.2)
Sick leave (%) (n = 776**)	346 (51 %)	56 (63 %)	1.7 (CI 1.1–2.6)	1.7 (CI 1–2.6)
Manual/chiropractic therapy	270 (23 %)	83 (46 %)	3 (CI 2.2–4.1)	3.4 (CI 2.3–4.8)
Physiotherapy	534 (46 %)	113 (63 %)	2 (CI 1.5–2.8)	1.6 (CI1.2–2.4)
Massage	350 (30 %)	67 (37 %)	1.4 (CI 1–1.9)	1.2 (CI 0.8–1.7)
Medication for LBP				
▪ No medication	320 (24 %)	25 (16 %)	0.5 (CI 0.34–0.9)	0.53 (CI 0.34–0.83)
▪ Non-opioids***	714 (61 %)	132 (75 %)	1.8 (CI 1.3–2.7)	1.6 (CI 1.1–2.5)
▪ Opioids	89 (7 %)	37 (21 %)	3.1 (CI 2–4.8)	2.7(CI 1.7–4.3)
Injection therapy	680 (58 %)	134 (75 %)	1.8 (CI 1.3–2.5)	2 (CI 1.4–2.9)
Electrotherapy	191 (16 %)	41 (23 %)	1.5 (CI 1.1–2.2)	1.5 (CI 0.9–2.3)
TENS***	89 (8 %)	35 (20 %)	3 (CI 2–4.7)	2.5 (CI 1.6–3.9)
Psychotherapy	75 (6 %)	20 (11%)	1.9 (CI 1.1–3.2)	1.3 (CI 0.8–2.4)
Back school	105 (9 %)	22 (12 %)	1.5 (CI 0.9–2.4)	1.3 (CI 0.8–2.2)
Hospital admission	66 (6 %)	20 (11 %)	2.1 (CI 1.3–3)	1.9 (CI 1–3.5)

Table legend text

* OR (= odds ratio) of a health care service utilisation in relation to acupuncture adjusted for gender, age group, pain chronicity, functional capacity, depression and radiation of pain below the knee

** Only working patients

*** Some Patients received opioid and non opioid-medication

**** TENS: transcutaneous electric nerve stimulation

CI: 95 % confidence intervals
